# Intramedullary spinal cord metastasis from esophageal squamous cell carcinoma: case report and literature review

**DOI:** 10.1186/s12883-023-03147-0

**Published:** 2023-03-08

**Authors:** Beiduo Shen, Zhaoyu Ba, Yufeng Huang, Desheng Wu

**Affiliations:** Department of Spine Surgery, Shanghai East Hospital, School of Medicine, Tongji University, 150 Jimo Road, 200120 Shanghai, China

**Keywords:** Intramedullary spinal cord metastasis, Cervical spine, Gadolinium enhanced magnetic resonance imaging, Esophageal squamous cell carcinoma, Case report

## Abstract

**Background:**

Intramedullary spinal cord metastasis (ISCM) of malignant tumors rarely happens. To the best of our knowledge, only five cases of ISCM from esophageal cancer have been reported in literature. We here report the sixth descripted case of ISCM from esophageal cancer.

**Case presentation:**

A 68-year-old male presented with weakness of right limbs and localized neck pain two years after diagnosed esophageal squamous cell carcinoma. The gadolinium enhanced Magnetic resonance imaging (MRI) of cervical spine showed a mixed-intense intramedullary tumor with typical more intense thin rim of peripheral enhancement in C4-C5. The patient died fifteen days after diagnosis of irreversible respiratory and circulatory failures. An autopsy was refused by his family.

**Conclusions:**

This case highlights the importance of gadolinium enhanced MRI for diagnosis in ISCM. We believe that early diagnosis and surgery for selected patients shows helpfulness to save their neurologic function and improve quality of life.

**Supplementary Information:**

The online version contains supplementary material available at 10.1186/s12883-023-03147-0.

## Background

Intramedullary spinal cord metastases (ISCMs) have very low incidence of only 8,5% among central nervous system metastases and nearly 3.5% of all spinal metastases [1–3]. They constitute 1–3% of all intramedullary tumors [4]. It is reported in the literatures that the majority of ISCMs derive from lung cancers and breast cancers [5, 6]. ISCM from esophageal cancer is even more rare, and only 5 described cases in 2 articles [5, 7] and 3 case reports [8–10] were found. We here report a rare cervical ISCM from esophageal squamous cell carcinoma.

## Case presentation

### History

A 68-year-old male presented localized pain of neck and ingravescent weakness in his right limbs for weeks in October 2019. This patient was diagnosed with esophageal squamous cell carcinoma in November 2017 by upper gastrointestinal endoscopy and received concurrent radiotherapy and chemotherapy for 34 times until March 2018. Paclitaxel and carboplatin were planned to be used. Between May 2018 and August 2019, the reexamination showed pulmonary and hepatic metastases. He underwent two times of docetaxel and six times of 5-fluorouracil and cisplatin accompany with 15 times of lung radiotherapy.

### Physical examination

Motor power of right upper limb is grade III, and grade II of right lower limb according to the British Medical Council grading system. The shallow sensation of the right side was weaker with no accurate sensory level. His bilateral patellar and ankle clonus and Babinski sign were positive. The power of his left limbs was nearly normal.

### Development, diagnosis and treatments

On day 2 of admission, the patient had difficulty urinating, numbness in the perineal area. Dyspnea was observed on that night. He was transferred to ICU for monitoring and life supports.

Between day 2 and day 5, respiratory rate and blood pressure fluctuations prevented the transfer to an MRI unit.

**Fig. 1:**
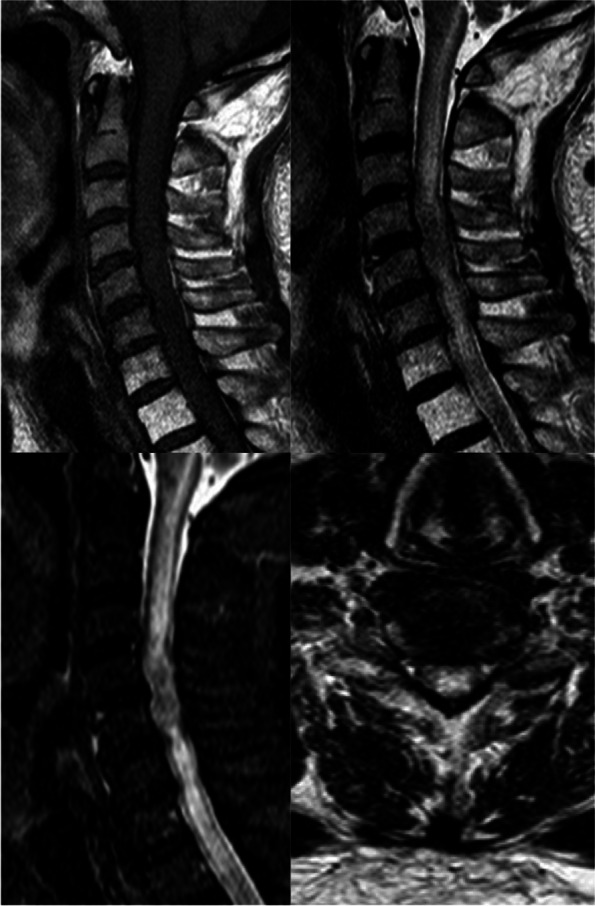
T2-weighted and STIR imaging demonstrated a hypo-isointense area in C4-5 with fusiform high intense of the surrounding spinal cord. The axial imaging suggested hyperintense intramedullary edema

On day 5, his vital signs were stable but neurologic function worsened. Reexamination demonstrated the motion of right lower limb was lost at grade I and grade III of the left. His shallow sensation decreased below the T6 level on the left and below T10 level on the right. Blood tests, head MRI and regular cervical spine MRI were completed. No sign of infection was observed(white blood cell: 4.38*10^9; C-reactive protein: 10.64 mg/L). The CEA (carcinoembryonic antigen) level was 6.92ng/ml (normal range < 5.2 ng/ml); and the NSE (neuron specific enolase) level was 32.4ng/ml (normal range < 16.3ng/ml). Head MRI indicated metastases in the left temporal lobe and the right parietal lobe, but respective locations had no connection with any of the neurologic deficits. T2-weight imaging of the cervical spine demonstrated a hypo-isointense area in C4-5 with fusiform high intense of the surrounding spinal cord (Fig. 1). Due to his rapid progression and poor general condition, methylprednisolone and mannitol were used; antibiotics and non-invasive respiratory support were provided in order to prevent secondary pneumonia.

Neurologists’ and radiologists’ consultation suspected the diagnosis of ISCM, but acute myelitis could not be excluded either.

The patient’s respiratory symptoms and the neurological function improved after treatments. The motor power of his right upper limb changed to nearly grade IV, and the distal motor power of his lower limbs turned to nearly V on the left and II on the right.

**Fig. 2:**
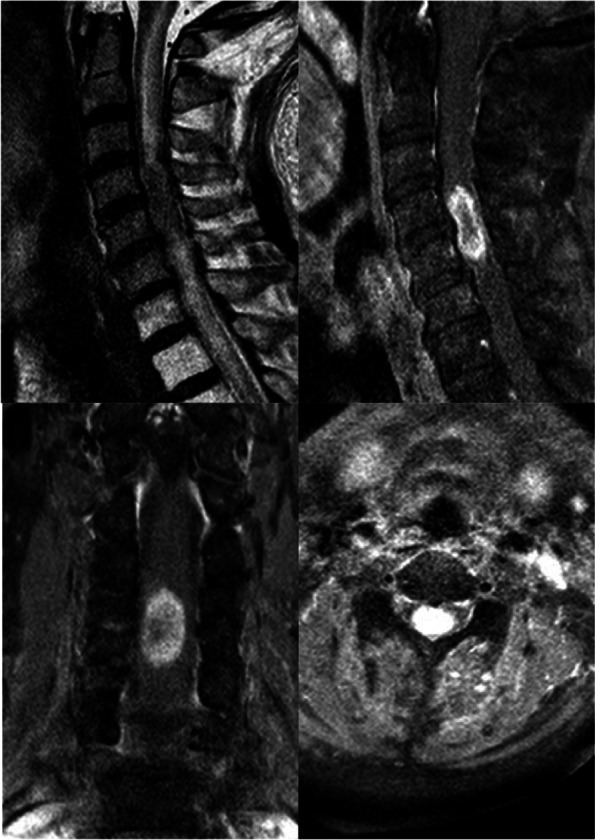
Sagittal, coronal and axial T1-weighted post-gadolinium enhanced imaging demonstrated a mixed-intense tumor with typical more intense thin rim of peripheral enhancement

The patient was transferred back to the general ward on the 11th day. A quick cerebrospinal fluid (CSF) examination and an enhanced cervical MRI was performed. There was no obvious abnormality in CSF examination, except for the mild elevation of glucose (4.930 mmol/L; normal range: 2.2–3.9 mmol/L) and protein (512 mg/L; normal range: 150–450 mg/L). However, a 26 mm*10mm*15mm intramedullary patchy intense tumor was found in C4-5 levels, surrounded by a more intense thin rim of peripheral enhancement in post-gadolinium T1 weighted MR images (Fig. 2). These results led to the diagnosis of ISCM.

Due to the multiple metastases and the instable condition of the patient, there was no indicates for surgery. The oncologists failed to perform a palliative radiotherapy because of a relapse of dyspnea and hypotension. As his family did not want any invasive respiratory supports, hospice care was offered.

### Outcome

The patient finally died of respiratory and circulatory failures one month after onset. An autopsy was refused by his family.

## Literature review and analysis

One case was reported by Grillo, A. et al. [9], which led to another two reported cases [8, 10]. And we discovered that Dam-Hieu, P. et al. included one patient from esophageal cancer in their retrospective study of 19 patients with ISCM in 2009 [5]. Kalayci, M. et al. may mention the first relative case in their illustrated review, but we could not find the origin article [7]. The 5 available corresponding outcomes were summarized in Table 1.

**Table 1:** A literature review of 5 available reported cases of ISCM from esophageal cancer, including the present report.

No. literature index	Age(years)/ gender	Time since PC diagnosis	Location	Presentation	Examinations	Other mets.	Treatments	Follow-up/ Outcome
1.Dam-Hieu, P. et al. [5]	61/M	1 month	C4	Quadriparesis; sphincter disorders	Enhanced-MRI	Brain	Surg.&RT	3 months/Died
2. Dalkilic, T. et al. [10]	80/M	NA	C4-7	Quadriparesis	Enhanced-MRI; Bone scan; PET/CT	No	Surg.&PRT	NA/improved
3.Nakamura, K. et al. [8]	78/M	2 months	C2	Diplegia; respiratory failure	Enhanced-MRI	lymph nodes	PRT	20days/Died
4. Grillo, A. et al. [9]	35/F	1 year	T9	Paraplegia; neurogenic bladder	Enhanced-MRI	Lymph nodes; vertebral bone; brain	PRT	2 months/Died
5.Shen. et al. (present report)	68/M	2 years	C4-5	Neck pain; right hemiplegia; dysuria; dyspnea;	Enhanced-MRI	Lung; liver; brain	Hospice care	1 month/Died

*Abbreviations*: *M* Male, *F* Female, *PC* Primary cancer, *mets* metastases, *MRI* Magnetic resonance imaging, *PET/CT* Positron emission tomography/ computed tomography, *surg*, surgery, *RT*, Radiotherapy, *PRT* Palliative radiotherapy, *NA* Not available.

The average age of the five patients was 64.4 years and only one female was reported. This female patient was relatively young (at the age of 35), and she was the only patient diagnosed within thoracic segment. The onset showed up variedly between 1 month to 2 years after primary cancer diagnosis. All the patients had enhanced-MRI examination and 80% of the patients(n = 4) were diagnosed based on the imaging. Brain metastasis appeared in 60% of the patients. Paralysis was observed in all patients, while most patients(n = 3,60%) also suffered from urinating issues. Respiratory failure happened in two patients. Two patients underwent surgical resections and both had improvement in neurological function after surgery. Four patients that received radiotherapy and had follow-up records ended up dead within 3 months.

## Discussion and conclusion

Intramedullary spinal cord metastases (ISCMs) are uncommonly observed. It is estimated at autopsy of 0.9–2.1% in patients with cancer [3, 11]. The latest article shows that 80% of the primary tumors in ISCM patients are from lung cancer, breast cancer, renal cell carcinoma and melanoma [6].

Three different routes of dissemination were hypothesized: hematogenous spread, meningeal carcinomatosis, and direct invasion. The most accepted is hematogenous spread which can explain why the metastasis may occur at any level of the spinal cord. Patients with ISCMs usually have a high rate of lung and intracranial metastases [12]. This theory seems to be conclusive, as our patients also fought for his pulmonary and hepatic metastases for years. Meanwhile, we also found metastases in his brain.

Weakness is the most common clinical symptom of ISCMs, while localized pain in back or neck, extremity pain, sensory loss, urinary incontinence, and Brown-Sequard syndrome are the other developments[6, 12, 13]. Patients with ISCMs usually experience a sudden onset and rapid progression of neurologic deficits which may help us to differ ISCM from primary intramedullary tumors [13].

The diagnosis of ISCMs mainly depends on the patient’s previous history and imaging findings. Gadolinium enhanced MR imaging is considered to be the most sensitive method to diagnose ISCM. Rykken et al. reported 2 enhancement features on MR imaging: a more intense thin rim of peripheral enhancement around an enhancing lesion (rim sign) and an ill-defined flame shaped region of enhancement at the superior/inferior margins (flame sign) [14]. It strongly suggests the diagnosis of ISCM, when either or both two signs are associated with an acute onset of symptoms in a cancer patient without previous evidence of spinal tumor [7, 10, 14]. However, even extremely rare, secondary intramedullary sarcomas of spinal cord following radiation therapy is reported in the literature [15]. There are no specific imaging criteria to differentiate intramedullary sarcomas from other varieties of spinal tumors or inflammatory masses. But they are more likely to be heterogeneous in density, with cystic and solid components [16]. We rule out the likelihood of secondary intramedullary sarcomas for the following reasons: (i) The neoplasm didn’t occurr within the radiation treatment field; (ii) there is no enough latency.

FDG-PET scan is considered to be useful in showing metabolic activities [7, 13]. Although our patient could not diagnose ISCM definitively for lack of autopsy, combined the patient’s rapid progression of neurological deficits with characteristic imaging evidence, let us have sufficient reasons to confirm it.

The presence of ISCM usually indicates high malignancy and poor prognosis. According to Goyal et al., the median survival of patients with ISCM was only 3.6 months. Age, sex, type of primary malignancy and the presence of brain or other systemic metastases did not influence survival [6]. By now, there is no guideline for the treatments. It suggests that the efficacy of chemotherapy is limited and shows no advantage in improving the survival rate [17, 18]. Scientists believe that the gold standard might still be radiotherapy, with or without steroids to reduce edema [12]. Meanwhile, surgery has become optional and can also be performed in selected cases. The principal objective should focus on the preservation of motor function [19]. Positive impacts of surgery on neurological outcome have been proved in patients with solitary intramedullary lesions. It may add the duration to nearly two times than conservative treatments, and quality of survival will improve simultaneously [6, 7, 12]. Radical resection of an ISCM is indicated in all patients who presents with rapid progressive neurological deficits [12]. However, patients in poor general health and having severe neurological deficits and multiple metastases represent obvious contraindications, especially in cases of complete paraplegia [5].

In conclusion, ISCM is an infrequent, deteriorating complication of malignancy. Early gadolinium enhanced MR imaging is particularly valuable for diagnosis of ISCM. Surgery seems to be helpful to save neurologic function and improve quality of life.

## Supplementary Information

**Additional file 1.**

## Data Availability

The datasets used and/or analysed during the current study are available from the corresponding author on reasonable request.

## Abbreviations

ISCM: Intramedullary spinal cord metastasis
MRI: Magnetic resonance imaging
ICU: Intensive Care Unit
CEA: carcinoembryonic antigen
NSE: neuron specific enolase
CSF: cerebrospinal fluid
FDG-PET: Fluorodeoxygluose Positron Emission Tomography
