# Supplementary material for: Intramedullary spinal cord metastasis from esophageal squamous cell carcinoma: case report and literature review
Source: BMC Neurol. 2023 Mar 8;23:100. doi: 10.1186/s12883-023-03147-0 (PMC9993626; doi:10.1186/s12883-023-03147-0)
Supplement: Supplementary file 1 — Additional file 1. [file 12883_2023_3147_MOESM1_ESM.pdf]

CARE case report guidelines

CARE Checklist of information to include when writing a case report

(cc) BY-NC-ND

✓

Topic	Item	Checklist item description	Reported on Line
Title	1	The diagnosis or intervention of primary focus followed by the words "case report"	✓
Key Words	2	2 to 5 key words that identify diagnoses or interventions in this case report, including "case report"	✓
Abstract (no references)	3a	Introduction: What is unique about this case and what does it add to the scientific literature?	✓
	3b	Main symptoms and/or important clinical findings	✓
	3c	The main diagnoses, therapeutic interventions, and outcomes	✓
	3d	Conclusion—What is the main "take-away" lesson(s) from this case?	✓
Introduction	4	One or two paragraphs summarizing why this case is unique (**may include references**)	✓
Patient Information	5a	De-identified patient specific information.	✓
	5b	Primary concerns and symptoms of the patient.	History
	5c	Medical, family, and psycho-social history including relevant genetic information	History
	5d	Relevant past interventions with outcomes	History
Clinical Findings	6	Describe significant physical examination (PE) and important clinical findings.	Physical examination
Timeline	7	Historical and current information from this episode of care organized as a timeline	Development, diagnosis and treatments
Diagnostic Assessment	8a	Diagnostic testing (such as PE, laboratory testing, imaging, surveys).	As above
	8b	Diagnostic challenges (such as access to testing, financial, or cultural)	As above
	8c	Diagnosis (including other diagnoses considered)	As above
	8d	Prognosis (such as staging in oncology) where applicable	As above
Therapeutic Intervention	9a	Types of therapeutic intervention (such as pharmacologic, surgical, preventive, self-care)	As above
	9b	Administration of therapeutic intervention (such as dosage, strength, duration)	As above
	9c	Changes in therapeutic intervention (with rationale)	As above
Follow-up and Outcomes	10a	Clinician and patient-assessed outcomes (if available)	Outcome
	10b	Important follow-up diagnostic and other test results	N/A
	10c	Intervention adherence and tolerability (How was this assessed?)	N/A
	10d	Adverse and unanticipated events	N/A
Discussion	11a	A scientific discussion of the strengths AND limitations associated with this case report	Discussion & Conclusion
	11b	Discussion of the relevant medical literature **with references**.	As above
	11c	The scientific rationale for any conclusions (including assessment of possible causes)	As above
	11d	The primary "take-away" lessons of this case report (without references) in a one paragraph conclusion	As above
Patient Perspective	12	The patient should share their perspective in one to two paragraphs on the treatment(s) they received	N/A
Informed Consent	13	Did the patient give informed consent? Please provide if requested	Yes ☑ No ☐
